# Pregnancy loss and role of infant HIV status on perinatal mortality among HIV-infected women

**DOI:** 10.1186/1471-2431-12-138

**Published:** 2012-08-31

**Authors:** Hae-Young Kim, Prisca Kasonde, Mwiya Mwiya, Donald M Thea, Chipepo Kankasa, Moses Sinkala, Grace Aldrovandi, Louise Kuhn

**Affiliations:** 1 Department of Epidemiology, Mailman School of Public Health, Columbia University, New York, NY, USA; 2 Gertrude H. Sergievsky Center, College of Physicians and Surgeons, Columbia University, 630 W 168th St, New York, NY, USA; 3 University Teaching Hospital, University of Zambia, Lusaka, Zambia; 4 Center for International Health and Development, Boston University of School of Public Health, Boston, USA; 5 Lusaka District Health Management Team, Lusaka, Zambia; 6 Department of Pediatrics, Children's Hospital Los Angeles, University of Southern California, Los Angeles, USA

**Keywords:** Perinatal mortality, Infant mortality, Risk factors, Adverse pregnancy outcome, HIV infection, Vertical transmission

## Abstract

**Background:**

HIV-infected women, particularly those with advanced disease, may have higher rates of pregnancy loss (miscarriage and stillbirth) and neonatal mortality than uninfected women. Here we examine risk factors for these adverse pregnancy outcomes in a cohort of HIV-infected women in Zambia considering the impact of infant HIV status.

**Methods:**

A total of 1229 HIV-infected pregnant women were enrolled (2001–2004) in Lusaka, Zambia and followed to pregnancy outcome. Live-born infants were tested for HIV by PCR at birth, 1 week and 5 weeks. Obstetric and neonatal data were collected after delivery and the rates of neonatal (<28 days) and early mortality (<70 days) were described using Kaplan-Meier methods.

**Results:**

The ratio of miscarriage and stillbirth per 100 live-births were 3.1 and 2.6, respectively. Higher maternal plasma viral load (adjusted odds ratio [AOR] for each log10 increase in HIV RNA copies/ml = 1.90; 95% confidence interval [CI] 1.10–3.27) and being symptomatic were associated with an increased risk of stillbirth (AOR = 3.19; 95% CI 1.46–6.97), and decreasing maternal CD4 count by 100 cells/mm^3^ with an increased risk of miscarriage (OR = 1.25; 95% CI 1.02–1.54). The neonatal mortality rate was 4.3 per 100 increasing to 6.3 by 70 days. Intrauterine HIV infection was not associated with neonatal morality but became associated with mortality through 70 days (adjusted hazard ratio = 2.76; 95% CI 1.25–6.08). Low birth weight and cessation of breastfeeding were significant risk factors for both neonatal and early mortality independent of infant HIV infection.

**Conclusions:**

More advanced maternal HIV disease was associated with adverse pregnancy outcomes. Excess neonatal mortality in HIV-infected women was not primarily explained by infant HIV infection but was strongly associated with low birth weight and prematurity. Intrauterine HIV infection contributed to mortality as early as 70 days of infant age. Interventions to improve pregnancy outcomes for HIV-infected women are needed to complement necessary therapeutic and prophylactic antiretroviral interventions.

## Background

In 2009, an estimated 1.4 million pregnant women were living with HIV in low- and middle-income countries. About 91% of those women in need of antiretroviral drugs for preventing mother-to-child transmission were in sub-Saharan Africa and 54% of women are estimated to have received the prophylactic drugs with recent scale-up [1]. Besides the risk of HIV transmission, maternal HIV infection is associated with increased risk of adverse pregnancy outcomes. For example, a meta-analysis of 31 studies showed almost a four-fold increased risk of stillbirth among HIV-infected compared to uninfected women [2], although some studies have not observed this [3,4].

Several studies in sub-Saharan African countries have also shown that infants born to HIV-infected women have a significantly increased risk of low birth weight (LBW) [4-7], preterm birth [5,6], neonatal mortality [7] and infant mortality [7-9]. Generally, markers of more advanced maternal disease, such as low CD4 cell counts and high plasma viral loads, are associated with these endpoints [9-11]. However, since these markers are also strongly related to HIV transmission [12-14], it is not clear whether infant HIV infection is a cause or a consequence of these adverse events. One study that separated HIV infections by their timing found that preterm birth was associated with intrapartum but not with intrauterine infection, suggesting prematurity as a risk factor for intrapartum infection rather than a cause of preterm delivery [15]. In some studies, intrauterine-infected children had smaller weight-for-gestation than expected, suggesting some growth consequences of early intrauterine HIV infection [16,17].

HIV-infected infants have a substantially higher risk of infant mortality compared to uninfected but exposed infants [18,19]. In addition, infants infected intrauterine or intrapartum have a worse prognosis than those infected through breastfeeding [19-21]. But it is unclear whether perinatally-acquired HIV infection will influence infant survival within the first months after birth. Since LBW and preterm birth are the major risk factors for neonatal and early infant mortality in all populations regardless of HIV status, separating out independent effects of these inter-related perinatal variables is complex and requires large sample sizes [22,23]. One large study in Tanzania found that among infants born to HIV-infected mothers, LBW, not infant HIV infection, was the strongest risk factor for neonatal mortality [21].

Here we investigate risk factors for miscarriage, stillbirth, neonatal mortality and early mortality among a cohort of pregnant HIV-infected women followed as part of a study in Lusaka, Zambia. We aim to identify independent contributions of advanced maternal disease and infant HIV status to adverse pregnancy and neonatal outcomes and focus on whether the timing of infant HIV infection influences the risk of these adverse outcomes.

## Methods

### Study design

We report a secondary analysis of data collected as part of the Zambia Exclusive Breastfeeding Study (ZEBS), a randomized clinical trial to examine whether exclusive breastfeeding to 4 months, followed by abrupt weaning, could reduce postnatal HIV transmission and child mortality. The study protocol and primary results have been reported elsewhere [24-26]. Briefly, a total of 1435 HIV-positive pregnant women were recruited at two antenatal care clinics in Lusaka between May 2001 and September 2004 if they intended to breastfeed, accepted to take single-dose nevirapine to prevent perinatal HIV transmission and consented to be assigned to either of the study and control groups. Antiretroviral therapy (ART) only became available in the public sector as the study was nearing completion. Written informed consent was obtained from all study participants. The study was approved by human subjects committees at all collaborating institutions.

### Study procedures

Socioeconomic and clinical data were collected at enrollment. Maternal blood samples were drawn at enrollment to test for CD4 cell counts (FACSCount; BD Immunocytometry Systems), hemoglobin level (Hemocue system), and plasma viral load (Roche Amplicor 1.5; Roche). All women with positive results of routine syphilis screening test (RPR) were treated with penicillin. Two subsequent antenatal visits were scheduled before delivery. Data on the circumstances of miscarriages and stillbirths were collected from medical records if available or from interview with the mothers. Gestational week at delivery was calculated from the estimated gestational week at enrollment, which was based on clinical examination (fundal height) and self-report of last menstrual period (LMP). In cases of discrepant gestational age estimates, the LMP date was used. Heel stick blood samples of infants were obtained at clinic visits scheduled at birth, 1 week and 5 weeks. DNA was extracted from each infant’s blood sample and tested for HIV by real-time polymerase chain reaction (PCR). All positive test results were confirmed by testing at least two samples. To rule out false negative test results due to an inadequate sample, amplification of the beta-globin gene was performed to ensure adequate cell numbers. Home visits were scheduled at 4 days after birth and 2, 3 and 7 weeks post-partum. Study participants who did not return for the scheduled antenatal or clinic visits were tracked and followed by home-visit teams.

### Study variables

The primary outcomes of interest were miscarriage, stillbirth, neonatal mortality (infant’s death <28 days) and early mortality (infant’s death <70 days). Miscarriage was defined as spontaneous abortion of pregnancy occurring before 24 gestational weeks, and stillbirth as fetal death occurring at 24 gestational weeks or later. Low birth weight (LBW) was defined as birth weight <2500 g, and preterm birth as having <34 weeks of gestation. This cut-off was selected since systematic under-estimation of gestational age by 2–3 weeks was indicated by the birth weight distribution. 30 infants did not have birth weight data mainly due to delivery at home. Their birth weights were predicted based on their weights measured at 1 week.

Maternal body mass index (BMI) at 1 month postpartum was used as a predictor since women were enrolled at different gestational weeks and often quite late in pregnancy. 123 women who missed the one month visit were included into models as a separate group. Breastfeeding was defined as having stopped only if cessation occurred before the illness preceding infant’s death or loss to follow up. Stopping breastfeeding was considered as a fixed-covariate and a time-dependent variable. After November 2003, cotrimoxazole prophylaxis was introduced for women with CD4 cell counts <200 cells/mm^3^ and the effects of cotrimoxazole were reported elsewhere [27]. If women experienced weight loss, >30 days of diarrhea, fever or cough in the six months prior to enrollment, or had history of thrush or tuberculosis, women were considered as being symptomatic according to World Health Organization criteria [28]. Further, each symptom was individually examined.

The timing of HIV transmission was stratified into three groups: 1) intrauterine transmission (IU) 2) intrapartum/early post-partum transmission (IP/ePP) and 3) no infection within 42 days after birth (NI). IU transmission was presumed if PCR results were positive within 3 days of birth. IP/ePP transmission was presumed if the PCR results were positive between 4 and 42 days after birth. If PCR results were negative at least for 42 days after birth, then the infants were presumed not to be infected within 42 days (NI). Mortality among the three groups with different timing of HIV transmission was compared. The cut-off point of early mortality was chosen at 70 days to measure the effect of IU and IP infection over the 6 weeks period after the neonatal period. Those with no PCR results were considered to be of unknown HIV status.

### Statistical analysis

Mantel-Haenszel relative risk (RR) of each factor on each outcome was examined and reported with 95% Confidence Intervals (CIs). For adjusted analysis, multivariable logistic regression was fitted. Variables with p-value < .10 in univariable analysis were included and were retained if significant (p-value <0.05) and their odds ratios (ORs) were reported with 95% CIs. Kaplan-Meier methods were used to calculate mortality rates for IU, IP/ePP and NI infants and Cox Proportional Hazards models to examine risk factors of neonatal mortality and early mortality [29]. Unadjusted and adjusted hazard ratios (HRs) were reported with 95% CIs. Continuous and categorical variables were compared across groups by two sided-Wilcoxon tests or t-tests and *χ*^2^ statistics, respectively. All statistical analyses were performed by SAS (version 9.2).

### Sensitivity analysis

Sensitivity analysis was performed to examine whether infants with unknown HIV status biased the results. First, we assumed that all infants with unknown status are IU, IP or NI. Next we estimated the probability of IU or IP transmission in the infants of unknown HIV status among those who died or who were lost to follow up separately based on a logistic regression model predicting transmission from maternal CD4 cell counts and plasma viral load. Finally, we calculated the expected neonatal and early mortality rates that would have occurred had these transmission rates occurred in each group of IU, IP and NI infants.

## Results

### Cohort description

Of 1435 HIV-positive women in the study, 11 women were not pregnant, 191 withdrew or were lost to follow up and four women died before delivery. Of 1229 women, 3.4% were enrolled during the first trimester and 62.7% during the second (median 26 weeks; IQR 20–31). There were 36 miscarriages and 30 stillbirths. Thus, 1163 women gave birth to 1185 live-born infants (26 non-singleton births including 21 pairs of twins both born alive). For 53 women, live-births were known to have occurred but no delivery or follow-up data were available. Compared to all 1163 live-born infants, the ratio of miscarriage and stillbirth per 100 live-births were 3.1 (95% CI 2.2–4.3) and 2.6 (95% CI 1.8–3.7), respectively. Among 1132 live-born infants with delivery records from 1110 mothers, 14.3% had LBW, and 16.3% were preterm. The probabilities of neonatal (<28 days) and early (<70 days) mortality were 0.043 (n = 47, 95% CI 0.032–0.057) and 0.063 (n = 67, 95% CI 0.050–0.079), respectively (Table 1). No woman with miscarriage or stillbirth had started ART and only 6 women started ART during pregnancy and 3 within 70 days post-partum.

**Table 1:** Frequencies of adverse pregnancy outcomes among 1229 HIV-infected pregnant women recruited in Lusaka, Zambia

	**No.**	**Ratio of event per 100 live-born infants**
**Miscarriage**	36	3.1
**Stillbirth**	30	2.6
**# of live-born infants with birth records**	N = 1132	**Rate**^**a**^**(95% CI)**
**Neonatal mortality (0–28 days)**	47	0.043 (0.032–0.057)
**Early mortality (0–70 days)**	67	0.063 (0.050–0.079)
**Birth Weight**^**b**^		**Percentage**
<1500 g	15	1.3
1500–1999 g	33	2.9
2000–2499 g	113	10.1
≥2500 g	963	85.7
Mean ± SD, g	2947.7 ± 531.7	
**Gestation**^**c**^		
<32 weeks	94	8.4
32–33 weeks	88	7.9
34–36 weeks	260	23.3
≥37 weeks	672	60.3
Mean ± SD, weeks	37.6 ± 4.05	

^a^ Calculated using Kaplan-Meier life table methods.

^b^ 8 infants are missing data on birth weight and weight at 1 week visit.

^c^ 18 infants are missing data on gestational weeks estimated at enrollment. Data suggested a systematic underestimation of gestation by 2–3 weeks. Thus we estimate that only 16.3% of births were preterm by conventional definitions of <37 weeks.

### Risk factors for miscarriage and stillbirth

Risk factors for miscarriage or stillbirth were examined (Table 2). CD4 cell counts <350 cells/mm^3^ and history of miscarriage were associated with an increased risk of miscarriage. For stillbirth, plasma viral load ≥50,000/ml and symptomatic HIV disease were significant risk factors as well as no previous pregnancies. In multivariable analysis, only CD4 cell counts <350 cells/mm^3^ was significantly associated with miscarriage. The odds ratio for decreasing CD4 cell counts by 100 cells/mm^3^ was 1.25 (95% CI 1.02–1.54, p = 0.03). For stillbirth, plasma viral load (adjusted OR [AOR] for each log10 increase in HIV RNA copies/ml = 1.90; 95% CI 1.10–3.27), being symptomatic (AOR = 3.19; 95% CI 1.46–6.97) and no previous pregnancy (AOR = 2.71; 95% CI 1.16–6.31) remained as significant risk factors.

**Table 2:** **Risk factors for miscarriage and stillbirth among a cohort of 1229 HIV-infected women**^**a**^

	**Ratio of miscarriages/Live births (per 100)**	**Relative risk (95% CI)**	**Ratio of stillbirths/Live births (per 100)**	**Relative risk (95% CI)**
***Maternal Health Factors***				
**CD4 cells/mm**^**3**^				
<350	24/652 (3.7)	**2.20 (1.03–4.69)**	19/647 (2.9)	1.44 (0.69–3.00)
≥350	9/538 (1.7)		11/540 (2.0)	
**Hemoglobin, g/dl**				
<10	12/352 (3.4)	1.34 (0.67–2.70)	7/347 (2.0)	0.73 (0.31–1.68)
≥10	21/826 (2.5)		23/828 (2.8)	
**Plasma viral load, copies/ml**				
≥50,000	19/540 (3.5)	1.62 (0.82–3.20)	19/540 (3.5)	**2.05 (0.99–4.28)**
<50,000	14/645 (2.2)		11/642 (1.7)	
**Clinical stage**				
Symptomatic	13/459 (2.8)	0.91 (0.47–1.78)	20/466 (4.3)	**3.12 (1.47–6.61)**
Asymptomatic	23/740 (3.1)		10/727 (1.4)	
***Socioeconomic and Clinical Factors***				
**Age, year**				
<20	3/117 (2.6)	0.89 (0.27–2.90)	5/119 (4.2)	1.73 (0.66–4.49)
20-30	25/867 (2.9)	1.0	21/863 (2.4)	1.0
>30	8/215 (3.7)	1.29 (0.59–2.82)	4/211 (1.9)	0.78 (0.27–2.25)
**Education**				
Some (0-8^th^ grade)	26/755 (3.4)	1.53 (0.74–3.14)	22/751 (2.9)	1.62 (0.73–3.60)
HS grade (≥ 9^th^ grade)	10/444 (2.3)		8/442 (1.8)	
**Electricity**				
No	22/718 (3.1)	1.05 (0.54–2.04)	20/716 (2.8)	1.33 (0.63–2.82)
Yes	14/481 (2.9)		10/477 (2.1)	
**Previous pregnancy**				
No	7/169 (4.1)	1.47 (0.66–3.30)	8/170 (4.7)	**2.19 (0.99–4.83)**
Yes	29/1030 (2.8)		22/1023 (2.2)	
**Previous miscarriages**				
Yes	7/111 (6.3)	**2.37 (1.06–5.28)**	5/109 (4.6)	1.99 (0.78–5.09)
No	29/1059 (2.7)		25/1084 (2.3)	
**Previous stillbirths**				
Yes	2/70 (2.9)	0.95 (0.23–3.87)	1/69 (1.5)	0.56 (0.08–4.06)
No	34/1129 (3.0)		29/1124 (2.6)	
**Rapid plasma reagin test**				
Positive	5/193 (2.6)	0.89 (0.35–2.29)	8/196 (4.1)	1.72 (0.78–3.81)
Negative	27/932 (2.9)		22/927 (2.4)	

^a^ All analyses are unadjusted.

### Risk factors for LBW and preterm birth

Risk factors for LBW and preterm birth are shown in Table 3. Higher plasma viral load, BMI <18.5 kg/m^2^ and swollen glands in more than one place were associated with both LBW and premature birth. In addition, women with CD4 cell counts <350 cells/mm^3^ had an increased risk of having LBW infants (RR = 1.57; 95% CI 1.16–2.12) compared to women with higher CD4 cell counts. Infants born to mothers with hemoglobin <10 g/dl, clinical symptoms or history of tuberculosis were more likely to be LBW. Less formal education was associated with preterm birth and women aged >30 years had a lower risk of preterm birth than women aged 20–30 years.

**Table 3:** **Risk factors for low birth weight (<2500g) and preterm birth among 1132 live-born infants**^**a**^

	**No. of LBW (%)**	**Relative risk (95% CI)**	**No. of preterm birth (%)**	**Relative risk (95% CI)**
***Infant Factors***				
**Gender**				
Male	79/587 (13.5)	0.88 (0.66–1.17)	97/583 (16.6)	1.03 (0.79–1.35)
Female	82/535 (15.3)		85/528 (16.1)	
***Maternal Health Factors***				
**CD4 cells/mm**^**3**^				
<350	105/611 (17.2)	**1.57 (1.16–2.12)**	108/608 (17.8)	1.24 (0.94–1.63)
≥350	56/510 (11.0)		72/503 (14.3)	
**Hemoglobin, g/dl**				
<10	66/330 (20.0)	**1.67 (1.25–2.23)**	54/325 (16.6)	1.04 (0.78–1.40)
≥10	93/778 (12.0)		123/773 (15.9)	
**Plasma viral load, copies/ml**				
≥50,000	96/505 (19.0)	**1.82 (1.36–2.45)**	94/500 (18.8)	**1.32 (1.01–1.72)**
<50,000	64/614 (10.4)		87/609 (14.3)	
**Clinical stage**				
Symptomatic	73/431 (16.9)	**1.33 (1.00–1.78)**	68/427 (15.9)	0.96 (0.73–1.26)
Asymptomatic	88/693 (12.7)		114/687 (16.6)	
**History of tuberculosis**				
Yes	28/106 (26.4)	**2.02 (1.42–2.88)**	23/105 (21.9)	1.39 (0.94–2.05)
No	133/1018 (13.1)		159/1009 (15.8)	
**History of having swollen glands**				
Yes	29/138 (21.0)	**1.57 (1.09–2.25)**	34/138 (24.6)	**1.62 (1.17–2.25)**
No	132/984 (13.4)		148/975 (15.2)	
**BMI, kg/m**^**2**^				
<18.5	37/150 (24.7)	**2.41 (1.71–3.39)**	34/146 (23.3)	**1.76 (1.25–2.47)**
≥18.5	88/858 (10.3)		112/845 (13.3)	
***Socioeconomic and Clinical Factors***				
**Age, year**				
<20	13/101 (12.9)	0.92 (0.54–1.57)	23/101 (22.8)	1.35 (0.92–2.00)
20–30	115/824 (14.0)	1.0	137/814 (16.8)	1.0
>30	33/199 (16.6)	1.19 (0.83–1.69)	22/199 (11.1)	**0.66 (0.43–1.00)**
**Education**				
Some (0–8^th^ grade level)	101/702 (14.4)	1.01 (0.75–1.36)	127/692 (18.4)	**1.41 (1.05–1.89)**
HS grade (≥9^th^ grade level)	60/422 (14.2)		55/422 (13.0)	
**Electricity**				
No	102/671 (15.2)	1.17 (0.87–1.57)	119/668 (17.8)	1.26 (0.95–1.67)
Yes	59/453 (13.0)		63/446 (14.1)	
**Food run out at least 1 day in last month**				
Yes	32/244 (13.1)	0.89 (0.62–1.28)	46/245 (18.8)	1.20 (0.89–1.62)
No	129/879 (14.7)		136/868 (15.7)	

^a^ All analyses are unadjusted.

In multivariable analysis, maternal plasma viral load (AOR for each log10 increase = 1.30; 95% CI 1.03–1.66), BMI <18.5 kg/m^2^ (AOR = 2.35; 95% CI 1.49–3.70), hemoglobin <10g/dl (AOR = 1.50; 95% CI 1.04–2.16), a history of swollen glands in more than one place (AOR = 1.64; 95% CI 1.02–2.63) or tuberculosis (AOR = 1.85; 95% CI 1.11–3.06) were each independently associated with a higher risk of LBW. For preterm birth, BMI <18.5 kg/m^2^ was the strongest risk factor (AOR = 1.96; 95% CI 1.27–3.04). History of swollen glands (AOR = 1.77; 95% CI 1.14–2.73) and less formal education (AOR = 1.42; 95% CI 1.00–2.02) were independently associated with preterm birth. Maternal age >30 years was still associated with reduced risk of preterm birth (AOR = 0.62; 95% CI 0.38–1.00).

### Risk factors for neonatal and early mortality

Risk factors for neonatal and early mortality are shown in Table 4. Preterm birth, LBW, APGAR scores and cesarean delivery were strongly associated with an increased risk of both neonatal and early mortality in univariable analysis. History of tuberculosis and >30 days of diarrhea were significant risk factors for both neonatal and early mortality. Relative risks of these factors were higher for neonatal mortality than for early mortality. Maternal health factors, namely CD4 cell counts <350 cells/mm^3^, hemoglobin <10 g/dl, higher plasma viral load, and BMI <18.5 kg/m^2^ were significantly associated with an increased risk of early mortality, but not with neonatal mortality.

**Table 4:** **Risk factors for neonatal mortality (<28 days) and early mortality (<70 days) among 1132 live-born infants**^**a**^

	**No.**	**Neonatal mortality**		**Early mortality**	
		**Mortality rate (n)**	**Hazard ratio (95% CI)**	**Mortality rate (n)**	**Hazard ratio (95% CI)**
***Infant Factors***					
**Sex**					
Male	592	0.042 (24)	1.00 (0.56–1.79)	0.067 (37)	1.18 (0.73–1.92)
Female	537	0.042 (22)		0.056 (29)	
**Gestation**					
Preterm	182	0.111 (19)	**3.87 (2.15–6.95)**	0.137(23)	**3.03 (1.83–5.03)**
Term	932	0.030 (27)		0.049 (43)	
**Birth weight, g**					
<2500	161	0.169 (26)	**8.85 (4.89–15.99)**	0.239 (36)	**8.69 (5.30–14.24)**
≥2500	963	0.020 (19)		0.031 (28)	
**APGAR score at 5 min**					
<7	17	0.647 (11)	**42.78 (20.89–87.59)**	0.718 (12)	**34.17 (17.78–65.66)**
≥7	995	0.027 (26)		0.045 (42)	
**APGAR score at 1 min**					
<7	30	0.580 (17)	**43.32 (22.78–82.39)**	0.627 (18)	**31.06 (17.55–55.00)**
≥7	989	0.023 (22)		0.041 (38)	
**Mode of delivery**					
C–section	35	0.205 (7)	**6.12 (2.74–13.66)**	0.239 (8)	**5.00 (2.39–10.46)**
Vaginal	1096	0.038 (40)		0.057 (59)	
***Maternal Health Factors***					
**CD4 cells/mm**^**3**^					
<350	616	0.052 (31)	1.62 (0.89–2.96)	0.084 (49)	**2.28 (1.33–3.92)**
≥350	513	0.032 (16)		0.037 (18)	
**Hemoglobin, g/dl**					
<10	334	0.062 (20)	1.76 (0.99–3.14)	0.089 (28)	**1.71 (1.05–2.78)**
≥10	782	0.036 (27)		0.053 (39)	
**Plasma viral load, copies/ml**					
≥50,000	510	0.055 (27)	1.66 (0.93–2.95)	0.085 (41)	**1.95 (1.19–3.18)**
<50,000	617	0.034 (20)		0.045 (26)	
**Clinical stage**					
Symptomatic	434	0.047 (20)	1.16 (0.65–2.07)	0.070 (29)	1.20 (0.74–1.95)
Asymptomatic	698	0.040 (27)		0.058 (38)	
**>30 days of diarrhea in the 6 months prior to enrollment**					
Yes	8	0.286 (2)	**7.18 (1.74–29.60)**	0.286 (2)	**5.21 (1.28–21.28)**
No	1124	0.041 (45)		0.061 (65)	
**History of tuberculosis**					
Yes	107	0.113 (12)	**3.32 (1.72–6.39)**	0.133 (14)	**2.55 (1.41–4.59)**
No	1025	0.035 (35)		0.055 (53)	
**BMI, kg/m**^**2**^					
<18.5	151	0.027 (4)	2.84 (0.85–9.42)	0.061 (9)	**2.86 (1.29–6.37)**
≥18.5	858	0.009 (8)		0.022 (18)	
**Maternal death before infant’s death**					
Yes	18	0.056 (1)	1.28 (0.18–9.25)	0.278 (5)	**4.85 (1.96–12.07)**
No	1114	0.043 (46)		0.059 (62)	
**Maternal death <70 days**					
Yes	18	0.282 (5)	**7.95 (3.14–20.09)**	0.521 (9)	**11.47 (5.68–23.18)**
No	1114	0.039 (42)		0.055 (58)	
**Stopped breastfeeding <70 days**					
Yes	36	0.112 (4)	**2.75 (0.99–7.66)**	0.260 (9)	**4.75 (2.35–9.58)**
No	1096	0.041 (43)		0.056 (58)	
***Socioeconomic and Clinical Factors***					
**Age, year**					
<20	103	0.080 (8)	**2.29 (1.05–4.99)**	0.104 (10)	**2.02 (1.02–4.02)**
20–30	828	0.038 (30)	1.0	0.056 (44)	1.0
>30	201	0.046 (9)	1.24 (0.59–2.60)	0.067 (13)	1.21 (0.65–2.24)
**Education**					
Some (0–8^th^ grade level)	708	0.040 (27)	0.81 (0.46–1.45)	0.061 (40)	0.90 (0.55–1.47)
HS grade (≥9^th^ grade level)	424	0.049 (20)		0.066 (27)	
**Electricity**					
No	676	0.043 (28)	1.00 (0.56–1.79)	0.068 (43)	1.22 (0.74–2.00)
Yes	456	0.043 (19)		0.055 (24)	
**Food run out at least 1 day in last month**					
Yes	247	0.033 (8)	0.72 (0.34–1.54)	0.060 (14)	0.93 (0.51–1.67)
No	884	0.046 (39)		0.064 (53)	
**# of children (<5 aged)**					
2+	163	0.032 (5)	0.71 (0.28–1.79)	0.046 (7)	0.69 (0.31–1.50)
0 –1	969	0.045 (42)		0.066 (60)	
**# of children (5–16 aged)**					
2+	360	0.026 (9)	0.49 (0.24–1.02)	0.037 (13)	**0.49 (0.27–0.90)**
0 –1	772	0.051 (38)		0.075 (54)	

^a^ All analyses are unadjusted.

Maternal death <70 days was associated with an increased risk of both neonatal and early mortality but many of these deaths occurred after infant death. Restricting only to maternal deaths occurring before infant death (18/1132; 1%), the association with early mortality was attenuated. Stopping breastfeeding <70 days was a significant risk factor for neonatal and early mortality. When fitted as a time-dependent variable, the association was further strengthened for both neonatal mortality (HR = 15.02; 95% CI 5.20–43.43) and early mortality (HR = 15.37; 95% CI 7.39–31.97). Infants born to mothers aged <20 years had a two-fold increased risk of neonatal and early mortality and having more than one child aged 5–16 years living in the household was associated with a lower risk of early mortality.

In multivariable analysis, LBW, stopping breastfeeding and young maternal age were significantly associated with both neonatal and early mortality. Preterm birth was only associated with neonatal mortality and low maternal CD4 cell counts (<350 cells/mm^3^) only with early mortality (Table 5, Model 1).

**Table 5:** **Multivariate analysis of risk factors for neonatal and early mortality**^**a**^

	**Neonatal mortality Adjusted HR (95% CI)**	**Early mortality Adjusted HR (95% CI)**
**Model 1**		
Low birth weight	6.10 (3.17–11.72)	7.52 (4.52–12.52)
Preterm birth	2.07 (1.08–3.94)	n/a
Stopping breastfeeding	8.61 (2.46–30.09)	8.36 (3.91–17.89)
CD4 count <350/mm^3^	n/a	1.82 (1.03–3.24)
History of tuberculosis	2.36 (1.14–4.92)	n/a
Maternal aged <20 years vs. 20–30 years	3.05 (1.3–6.81)	2.58 (1.24–5.38)
Having 2+ children age 5–16 years	n/a	0.44 (0.24–0.82)
**Model 2**		
HIV status		
IU transmission	2.00 (0.58–6.89)	2.76 (1.25–6.08)
IP transmission	0.77 (0.10–5.80)	1.87 (0.56–6.16)
Low birth weight	6.69 (2.68–16.71)	8.11 (4.33–15.21)
Preterm birth	2.75 (1.13–6.69)	n/a
Stopping breastfeeding^b^	5.50 (1.20–25.17)	9.84 (4.36–22.20)
Having 2+ children aged 5–16 years	n/a	0.46 (0.22–0.96)

^a^ Adjusted hazard ratios in models including all covariates as shown.

^b^ Stopping breastfeeding was fitted as a time-dependent covariate.

### Effects of infant HIV status

Among the 1132 infants, 50 infants had no HIV test results. Of 1082 infants with a known HIV status, 64 infants (5.9%) were IU infected. Another 64 infants (5.9%) were IP/ePP infected including 10 infants with indeterminate HIV status at birth but tested HIV-positive by 42 days. 863 infants were followed up and not infected at least for 42 days. 69 infants were lost to follow up but confirmed HIV-negative prior to 42 days and another 22 infants were HIV-negative at birth or 1 week but died before 42 days. These were included as NI infants, giving a total of 954 NI infants (88.2%).

Neonatal mortality rates were 5.0 per 100 live-born infants (95% CI 1.7–14.8) in IU infants, 1.6 (95% CI 0.2–10.6) in IP/ePP infants and 2.2 (95% CI 1.3–3.3) in NI infants. By 70 days after birth, early mortality increased to 13.7 per 100 (95% CI 7.1–25.5) in IU infants, 4.8 (95% CI 1.6–14.1) in IP/ePP infants and 3.5 (95% CI 2.5–5.0) in NI infants. Infants with IU infection had a non-significant elevation in mortality by 28 days compared to NI infants (HR = 2.29; 95% CI 0.68–7.70). The risk associated with IU infection increased when mortality through 70 days was considered (HR = 3.93; 95% CI 1.81–8.53). Model 2 in Table 5 shows the associations between IU and IP/ePP transmission and neonatal and early mortality in multivariable model. IU infection remained significantly associated with early mortality (AHR = 2.76; 95% CI 1.25–6.08) after adjustment for LBW, preterm birth, stopping breastfeeding, and having more than a child aged 5–16 years. IP/ePP infection was not significantly associated with early mortality and cannot be directly examined in relation to neonatal mortality since survival to a subsequent test was a component of the definition of intrapartum infection. Major causes of neonatal and early mortality were similar among the three groups; pneumonia, acute diarrhea and septicemia.

When we examined the mortality between 28 and 70 days, the risk of the mortality among IU infants was further elevated compared to NI infants (HR = 6.82; 95% CI 2.40–19.35) and still significant after adjustment for LBW and cessation of breastfeeding (AHR = 6.36; 95% CI 2.22–18.27). Non-significant increase in mortality was observed among IP/ePP infants (HR = 2.34; 95% CI 0.52–10.43).

To consider whether risk factors for neonatal and early mortality were modified by infant HIV status, we stratified the analysis by infant HIV status. In multivariable model restricting to 128 infected infants, only LBW was associated with neonatal mortality (HR = 16.63; 95% CI 1.73–160.16) and LBW (AHR = 16.13; 95% CI 4.23–61.48) and stopping breastfeeding (AHR = 8.15; 95% CI 1.62–40.92) with early mortality. Among 954 uninfected infants, LBW (AHR = 6.99; 95% CI 2.61–18.71) and preterm birth (AHR = 2.64; 95% CI 1.00–6.99) were significantly associated with neonatal mortality. LBW (AHR = 6.47; 95% CI 3.13–13.34) and stopping breastfeeding (AHR = 9.52; 95% CI 3.65–24.85) were significantly associated with early mortality as seen in whole population.

### Sensitivity analysis

The 50 infants with unknown HIV status had lower birth weights and higher mortality rates than IU, IP and NI infants. 23 infants (46%) died by 28 days and 24 infants (48%) by 70 days. To determine the extent of bias, we performed a sensitivity analysis including these infants under different assumptions (Table 6).

**Table 6:** Sensitivity analyses for the 50 infants with unknown HIV status

**MTCT**	**No.**	**Neonatal morality**			**Early mortality**		
		**Mortality Rate (n)**	**Crude HR (95% CI)**	**Adjusted HR(95% CI)**^**e**^	**Mortality Rate (n)**	**Crude HR (95% CI)**	**Adjusted HR(95% CI)**^**f**^
**Model 1**^**a**^							
IU	64	0.050 (3)	1.09 (0.34–3.50)	**0.97 (0.30–3.18)**	0.137 (8)	2.26 (1.08–4.73)	**1.65 (0.77–3.50)**
IP/ePP	64	0.016 (1)	0.35 (0.05–2.51)	**0.39 (0.05–2.85)**	0.048 (3)	0.78 (0.25–2.50)	**1.11 (0.35–3.58)**
NI at least for 42 days	1004	0.044 (43)	1.0		0.059 (56)	1.0	
**Model 2**^**b**^							
IU	114	0.268 (26)	14.82 (8.25–26.60)	**12.03 (6.45–22.42)**	0.347 (32)	12.65 (7.73–20.72)	**8.15 (4.83–13.74)**
IP/ePP	64	0.016 (1)	0.73 (0.10–5.40)	**0.73 (0.10–5.47)**	0.048 (3)	1.34 (0.41–4.39)	**1.70 (0.52–5.58)**
NI at least for 42 days	954	0.022 (20)	1.0		0.035 (32)	1.0	
**Model 3**^**c**^							
IU	64	0.050 (3)	2.28 (0.68–7.68)	**1.88 (0.55–6.43)**	0.137 (8)	3.89 (1.79–8.44)	**2.70 (1.23–5.91)**
IP/ePP	114	0.238 (24)	13.15 (7.25–23.84)	**11.65 (6.23–21.79)**	0.275 (27)	9.92 (5.93–16.58)	**9.61 (5.58–16.54)**
NI at least for 42 days	954	0.022 (20)	1.0		0.035 (32)	1.0	
**Model 4**^**d**^							
IU	67	0.080 (5)	1.94 (0.77–4.93)	**1.66 (0.65–4.29)**	0.164 (10)	2.98 (1.52–5.87)	**2.09 (1.05–4.17)**
IP/ePP	67	0.045 (3)	1.13 (0.35–3.65)	**0.82 (0.20–3.41)**	0.076 (5)	1.39 (0.56–3.48)	**1.55 (0.56–4.32)**
NI at least for 42 days	998	0.040 (39)	1.0		0.055 (52)	1.0	

^a^ Assume all of the 50 infants as NI at least for 42 days.

^b^ Assume all of the 50 infants infected by IU transmission.

^c^ Assume all of the 50 infants infected by IP/ePP transmission.

^d^ HIV status of the 50 infants was imputed based on the predicted probability of HIV transmission.

^e^ Adjusted for stopping breastfeeding, LBW, and preterm birth.

^f^ Adjusted for stopping breastfeeding, LBW, and 2+ children (5–16 aged).

The predicted probability of HIV transmission was 6.8% for IU and 6.9% for IP in the 24 infants who died before 70 days and 4.4% for IU and 4.0% for IP in the 26 infants lost to follow up. Assuming these transmission rates among the infants with unknown HIV status, we calculated neonatal mortality rates of 7.3% in IU, 3.9% in IP and 4.1% in NI groups. Early mortality rates were 15.6% in IU, 7.1% in IP and 5.5% in NI groups among the 1132 infants. After this imputation, the adjusted HRs of neonatal mortality and early mortality among IU transmitted infants were 1.66 (95% CI 0.65–4.29) and 2.09 (95% CI 1.05–4.17) compared to NI infants, respectively (Model 4). This is similar to the results excluding the infants of unknown HIV status suggesting that the original approach was reasonable.

## Discussion

This study documents that women with advanced HIV disease are not only more likely to transmit HIV but also have an increased risk of miscarriage and stillbirth and infants born to these mothers have an increased risk of neonatal and early mortality. High rates of neonatal and early mortality are primarily attributable to low birth weight and preterm birth, both also increased among women with advanced disease. It is only after the neonatal period that the infant’s HIV status begins to make a significant contribution to excess mortality.

In this study, the risks of miscarriage and stillbirth were 31 and 26 per 1000 live-born births, respectively. From another cohort study in Lusaka, Zambia, the estimated rate of stillbirth was 21 per 1000 live-born births where 75.2% of the participating women were HIV-uninfected [30]. We found that among HIV-infected women, miscarriage was associated with CD4 cell counts <350 cells/mm^3^. This result is similar to other studies [31,32]. In a South African cohort study, women with CD4 cell counts <250 cells/mm^3^ had a two-fold increased risk of the adverse outcome (antenatal heath, miscarriage or stillbirth) compared to those with CD4 cell counts >500 cells/mm^3^[31]. Another study in India reported that symptomatic women had a significantly increased rate of miscarriage, compared to asymptomatic women [32].

In our study, high plasma viral load was significantly associated with stillbirth among HIV-infected women. Many studies have reported increased risk of stillbirth in HIV-infected women compared to HIV-negative women [2], but only two studies have examined risk factors associated with stillbirth among HIV-infected pregnant women [3,33]. A study in Tanzania found that elevated CD3 count, but not other markers such as CD4 or CD8 counts, was associated with stillbirth [33]. In the cohort conducted in four cities in sub-Saharan Africa, the risk of stillbirth was correlated with decreasing CD4 count [3] but not with plasma viral load categorized into ≥100,000 copies/ml or less. In our analysis, the mean baseline plasma viral load of mothers who delivered stillbirths was significantly higher (median value 76,560 vs. 39,783 copies/ml, p = 0.01) thus fitting it as a continuous variable (log scale) seemed to be more accurate. Furthermore, symptomatic women had a more than a three-fold increased risk of stillbirth compared to asymptomatic women, suggesting that maternal disease progression may play an important role in perinatal outcomes.

Some previous studies have tried to document HIV status of pregnancy losses [34-36]. One study which did so by *in situ* hybridization showed that 60% of the spontaneous fetal losses (6/10) were associated with HIV transmission to the fetus [35]. It is possible that advanced maternal disease might have increased pregnancy losses via intrauterine transmission [37,38] but we could not directly examine such mechanism. Since ART only became available towards the end of the study, 52 out of 66 women (79%) who had miscarriages or stillbirths should have received ART under the current WHO guidelines but did not. Although certain ART regimens may increase the risk of adverse pregnancy outcomes [39,40], it also reduces the risk of perinatal transmission and improves maternal health [41]. Timely ART for HIV-infected pregnant women may result in a net improvement in these perinatal outcomes.

Our data indicate that HIV infection acquired during pregnancy begins to contribute to excess mortality as early as 70 days but mostly only after the neonatal period, independent of LBW and early cessation of breastfeeding. After the neonatal period, IU-infected infants had more than a six-fold increased risk of mortality compared to NI infants through 70 days. Prior studies have not as precisely considered the timing of early mortality of infected infants [19,20] nor separated IU transmission from IP/ePP transmission [21]. Some studies have found IU-infected infants to have onset of symptoms in first few months of life and faster disease progression than those infected at delivery or later [15,42,43]. Despite recent scale-up, only 15% of HIV-exposed infants had access to early infant diagnosis and one-third of infants diagnosed of HIV did not receive appropriate antiretroviral treatment in 2011 [44]. Our results re-emphasize the crucial role of early infant diagnosis in order to initiate therapy for infected infants given their rapid progression [45,46].

LBW was previously associated with infant death between 8 weeks and 6 months of life [9], post-neonatal death (29–365 days) and infant mortality [21] among HIV-infected children as well as among HIV-exposed uninfected [22]. Among perinatally-infected infants, the effect of LBW on infant survival dominates the neonatal period but persists thereafter even once HIV-related disease contributes to infant mortality. In our study, high maternal viral load and low hemoglobin were the most significant predictors of LBW and preterm birth as observed in other studies [4,16,47]. Advanced maternal disease seems to indirectly affect early infant survival via the risk of LBW and premature birth. Thus to complement antiretroviral interventions, other interventions such as providing cotrimoxazole [27] or multivitamin supplements during pregnancy [48] may need to be considered to improve overall birth outcomes.

Early breastfeeding cessation was a significant risk factor for both neonatal and early mortality. Breastfeeding has a significant role in infant survival for all infants including those born to HIV-infected women [20,25,26]. Maternal death was also related to infant mortality and is closely related to cessation of breastfeeding. However, we found breastfeeding to be a strong protector of infant survival independent of confounding effects of maternal health and survival.

Some social factors significantly affected infant survival. Infants with more than one sibling aged 5–16 years had decreased rates of early mortality. This may reflect families with greater birth spacing or substantial care provided by an older sibling. Teenage pregnancy was associated with an increased risk of neonatal and early mortality. In the general population, some studies have reported adverse associations between teenage pregnancy and perinatal mortality, largely attributable to preterm birth [49-51], while others have not [52]. In our study, maternal age <20 years was associated with neonatal mortality independent of preterm birth.

The study has several limitations. First, most of study participants were enrolled into the study during second or third trimester thus miscarriages and stillbirths occurring in earlier gestational ages are excluded resulting in underestimated rates of these outcomes. Our study thus has limited capacity to comment on the predictors of early pregnancy loss. However, most early pregnancy loss is due to chromosomal abnormalities and is unlikely to be influenced by maternal HIV status [53]. Second, distinguishing between miscarriage and stillbirth is difficult particularly with inaccuracies in estimates of gestational age. Nevertheless, similar factors were associated with both of these adverse outcomes hence the consequence of misclassification seems to be limited. Third, HIV status could not be determined for some infants. However, we examined the extent of bias in sensitivity analyses which suggested that these exclusions did not appreciably affect the results. Finally, the cut-off of 70 days might have been too early to see any adverse effects of IP/ePP infection but this analysis aimed to examine how rapidly intrauterine and perinatally-acquired HIV infection affect infant survival.

## Conclusion

Women with more advanced HIV disease had a higher risk of pregnancy loss and perinatal mortality. Advanced maternal HIV disease affected infant health and survival directly via the risk of HIV transmission and indirectly via LBW and prematurity. The effect of intrauterine infection in the infant was apparent by 70 days but excess neonatal mortality was primarily attributed to LBW and preterm birth independent of infant HIV status. Interventions to improve maternal health as well as pregnancy outcomes for HIV-infected women may be necessary to complement the currently recommended prophylactic and therapeutic antiretroviral regimens.

## Competing interests

The authors declare that they have no competing interests.

## Authors contributions

The authors’ responsibilities were as follows - LK, DMT, and GA: conceptualization and design; MS, CK, MM, DMT, and LK: study implementation and management; GA: laboratory assays; HYK and LK: analysis and statistics; and HYK and LK: draft of the manuscript. All authors: interpretation of data and revision of manuscript for critical content. All authors read and approved the final manuscript.

## Pre-publication history

The pre-publication history for this paper can be accessed here:

http://www.biomedcentral.com/1471-2431/12/138/prepub
